# Microbiome Dynamics in Early Life Stages of the Precious Mediterranean Red Coral *Corallium rubrum*

**DOI:** 10.1111/1758-2229.70127

**Published:** 2025-06-17

**Authors:** R. Tignat‐Perrier, L. Bramanti, B. Giordano, J. A. J. M. van de Water, E. Manea, D. Allemand, C. Ferrier‐Pagès

**Affiliations:** ^1^ Unité de Recherche sur la Biologie des Coraux Précieux CSM ‐ CHANEL Centre Scientifique de Monaco Monaco; ^2^ Coral Ecophysiology Laboratory Centre Scientifique de Monaco Monaco; ^3^ CNRS‐Sorbonne Université, Laboratoire d'Ecogéochimie des Environnements Benthiques, LECOB, Observatoire Océanologique de Banyuls‐sur‐mer Banyuls‐sur‐mer France; ^4^ Department of Life and Environmental Sciences University of Cagliari Cagliari Italy; ^5^ Department of Estuarine & Delta Systems Royal Netherlands Institute for Sea Research Yerseke the Netherlands; ^6^ Centre Scientifique de Monaco Monaco

**Keywords:** *16S rRNA* gene sequencing, bacterial communities, coral larvae, holobiont, microbiota, octocorals

## Abstract

Microbial colonisation in the early life stages of corals plays a critical role in their fitness, but the mechanisms by which microbial symbionts are acquired—whether from parental colonies, the environment, or both—remain poorly understood, particularly in octocorals. Although they are the second most abundant coral group on tropical reefs and contribute significantly to the structural complexity of Mediterranean coral reefs, their microbial dynamics are largely unexplored. In this study, we investigated the acquisition of the bacterial microbiota in the red coral *Corallium rubrum* , a precious coral. By analysing the composition of the bacterial community at different early life stages, including newly released larvae, 5‐ and 10‐day‐old larvae, 3‐month‐old settlers, 1‐year‐old recruits and 3‐year‐old juveniles, we are gaining new insights into the development of its microbiome. Using a direct PCR‐based *16S rRNA* metabarcoding approach, we performed high‐resolution microbiome analyses at the level of individual larvae and settlers. Our results show that the bacterial microbiota of *C. rubrum* matures after the first year of life. Notably, dominant symbionts, such as *Spirochaetaceae* and *BD72BR169 Gammaproteobacteria,* were absent in larvae, settlers and recruits, suggesting that they were likely acquired horizontally from the environment. These findings improve our understanding of the microbial colonisation and development of *C. rubrum* and shed light on the potential role of its bacterial community in holobiont function.

## Graphical abstract

The bacterial microbiota of the precious red coral *Corallium rubrum* matures after the first year of life. Specifically, the dominant symbionts of the adult colonies such as Spirochaetaceae were absent in larvae, settlers and recruits, suggesting that they were likely acquired horizontally from the environment between 6 months and 3 years of age.

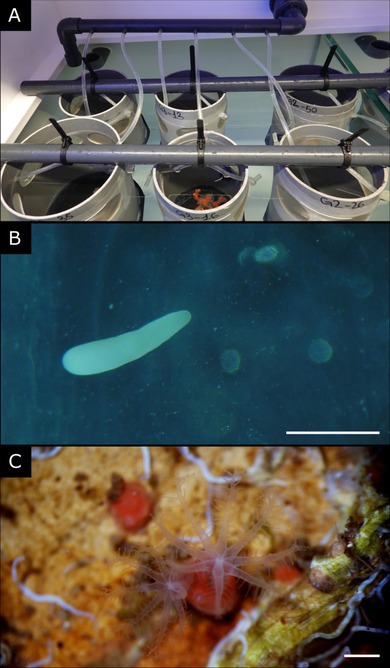

## 1 Introduction

The important role of microbial communities in shaping the evolution and biology of animals is becoming increasingly clear. They influence their development from the earliest stages of life to adulthood (O'Neill et al. 2020; Bemark et al. 2024; Diez‐Méndez et al. 2023). For example, early interactions with associated and surrounding microbial communities significantly influence the development of an animal's immune system (O'Neill et al. 2020; Bemark et al. 2024; Diez‐Méndez et al. 2023; Chen et al. 2020). In tropical scleractinian corals, studies have also demonstrated that bacteria play a major role in coral settlement (Ritson‐Williams et al. 2014; Tebben et al. 2011; Webster et al. 2004; Dobretsov and Rittschof 2020), a key process of reef resilience. For example, crustose coralline algae (CCA) can attract coral larvae and promote their settlement and metamorphosis through chemical (Jorissen et al. 2021; Gómez‐Lemos et al. 2018; Kitamura et al. 2009; Tebben et al. 2015), spectral (Foster and Gilmour 2016) and microbiological cues (Jorissen et al. 2021; Petersen et al. 2021; Siboni et al. 2020). The interaction of corals with their associated or resident microbes is also of utmost importance for the development of essential symbiotic relationships. The resident microbiota is established during the host's early life stages, undergoing gradual refinement until it reaches maturity. The initial acquisition of microbial symbionts occurs either through maternal inheritance (i.e., vertical transmission) or from the environment (i.e., horizontal transmission) (Ali et al. 2019; Chamberland et al. 2017; Leite et al. 2017; Quigley et al. 2018; Zanotti et al. 2020). In horizontal transmission, free‐swimming coral larvae, settlers or recruits establish symbioses with specific microbes that are present in the surrounding environment, such as the sediment (Ali et al. 2019; Nitschke et al. 2016). Vertical transmission ensures the maintenance of mutualistic relationships, particularly when specific symbionts are important for larval development. For example, the vertical transmission of nitrogen‐fixing *Rhizobiales* is essential for the early development of the tropical coral *Acropora millepora* (Lema et al. 2014). However, under certain conditions, vertical transmission can have negative effects on coral larvae. Inherited microalgae from the *Symbiodiniaceae* family, for example, have been shown to reduce the survival of larvae in some broadcast‐spawning tropical corals, due to the oxidative stress caused by naturally high UV levels (Baird et al. 2021).

While the interactions between microorganisms and the early life stages of scleractinian corals have been investigated (Ali et al. 2019; Baird et al. 2021; Damjanovic et al. 2019), no study has yet been conducted on other coral taxa, such as octocorals. However, octocorals are one of the most abundant groups of macrobenthic animals on temperate and tropical reefs. In the Mediterranean Sea, octocorals are the main engineer species of the coralligenous ecosystems (Orejas et al. 2022; Paoli et al. 2017; Rossi et al. 2017) forming the so‐called marine animal forests (MAFs) (Rossi et al. 2017), which provide an important habitat for many species (Paoli et al. 2017; Rossi et al. 2017; Ponti et al. 2019). Recently, Mediterranean octocorals have suffered significant losses due to direct human activities (e.g., fishing, water pollution) and global climate change (e.g., ocean warming) (Grenier et al. 2023; Estaque et al. 2023; Garrabou et al. 2009; Garrabou et al. 2022; Verdura et al. 2019). In particular, the unprecedented severity of marine heatwaves in 2022 and 2023 has been associated with disease outbreaks and mass mortality of octocorals. These mass mortality events have raised serious concerns about the recovery capacity of populations located in the first 40 m depth, which are the most vulnerable to the effects of marine heatwaves (Grenier et al. 2023; Estaque et al. 2023). Therefore, optimal reproductive performance of the surviving colonies and a high survival rate of the offspring are crucial for the maintenance of Mediterranean MAFs.

Given the importance of microbial symbionts in coral health, we investigated the acquisition of these symbionts by the Mediterranean coral *Corallium rubrum* , commonly known as the red coral or precious coral, which has been classified as an endangered species on the Red List by the International Union for Conservation of Nature (Otero et al. 2017). The red coral is a slow‐growing and long‐lived organism. Its hard, red skeleton has been of cultural, religious and economic importance since ancient times and continues to be used in jewellery today. Previous studies have shown that adult colonies are associated with a species‐specific bacterial community, dominated by the family *Spirochaetaceae*, that is highly stable both temporally and geographically (van de Water et al. 2016; van de Water, Voolstra, et al. 2018). In addition, *C. rubrum* was recently found to have evolutionary, cophylogenetic relationships with several bacterial symbionts (Prioux et al. 2024), including *Spirochaeta*, which may be implicated in the red colour of this coral (van de Water et al. 2024). However, the mechanisms of acquisition of this bacterial community are unknown. *C. rubrum* is a gonochoric internally brooding species that releases mature lecithotrophic ciliated larvae (planulae) between late July and early August (Bramanti et al. 2005; Santangelo et al. 2004). Based on the microbiome stability and reproductive strategy (i.e., brooding species that undergoes internal fertilisation), we hypothesised that the bacterial symbionts are vertically transmitted (van de Water, Allemand, et al. 2018). To test this hypothesis, we analysed the bacterial community composition at different life stages (newly released larvae, 5‐ and 10‐day‐old larvae, 3‐month‐old settlers, 1‐year‐old recruits and 3‐year‐old juvenile colonies), applying a novel direct PCR approach which allows us to examine the composition of the bacterial communities associated with a single coral larva. By analysing the early life stages of the red coral, we aimed to determine the timing at which *C. rubrum* acquires its microbiota, specifically the dominant symbionts *Spirochaetaceae*, and to characterise the symbiont reservoir (i.e., mother colony, surrounding adult colonies and environment).

## 2 Material and Methods

### 2.1 Biological Material

**FIGURE 1:**
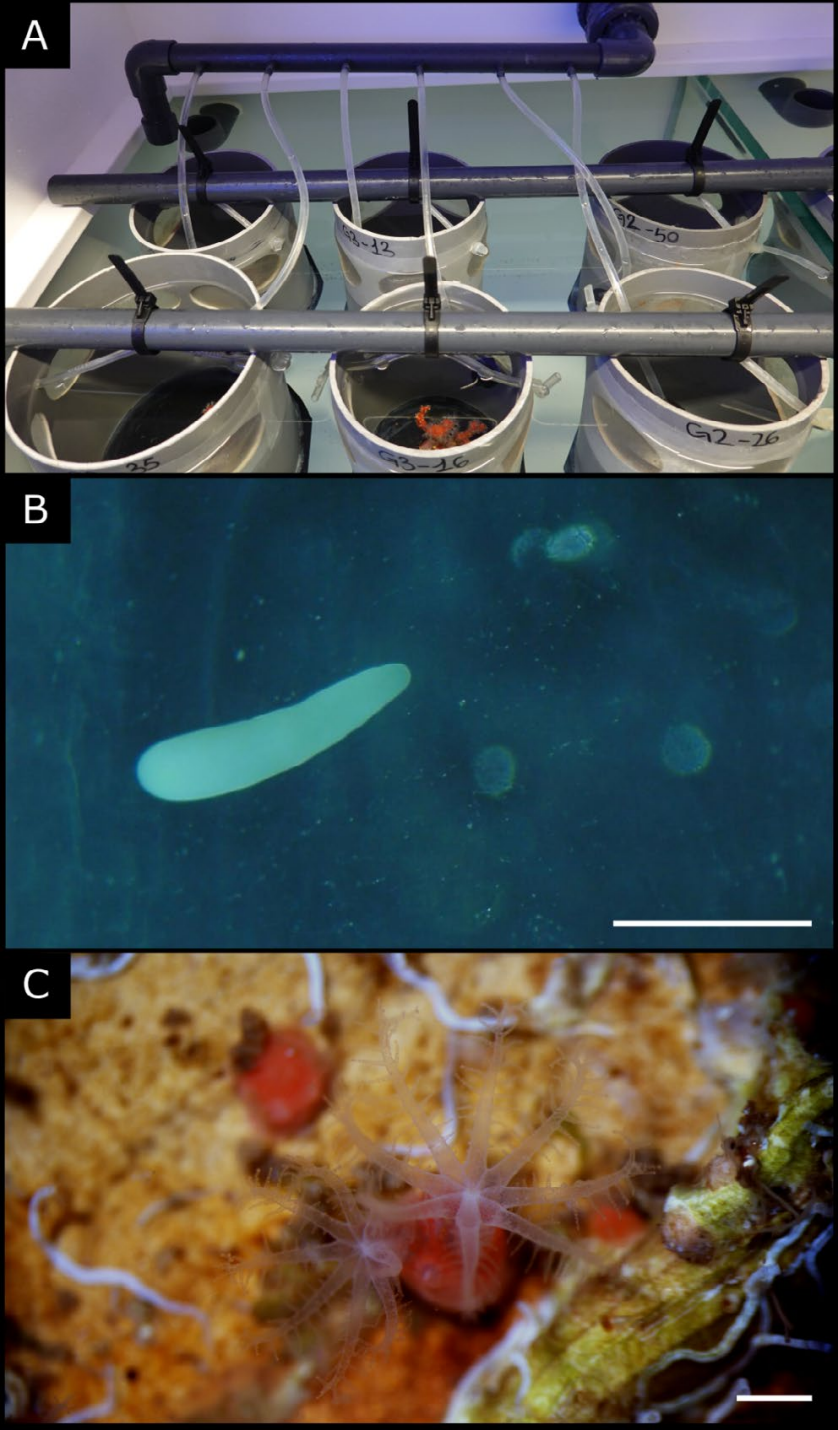
Biological material. (A) the laboratory system set up to collect larvae released by each mother colony, (B) a *C. rubrum* larva (planula) and (C) a 1‐year‐old recruit with its two polyps open observed under a binocular microscope. White lines correspond to 1 mm (photo credits: Bruna Giordano).

At the beginning of July 2022, a large multi‐branched colony (Colony 1) was found on the sea bottom in the Cerbère/Banyuls Marine Reserve at a depth of 30 m and was transferred to the laboratory aquarium of the Observatoire Océanographique of Banyuls‐sur‐mer in line with the recovery protocol established with the Marine Protected Area managers. Three smaller multi‐branched colonies (colonies 2, 3 and 4) were collected in the same area and at the same depth, but from a system of artificial caves deployed for a *C. rubrum* restoration project (Giordano 2024; Giordano 2025). Coral collection was authorised by the Direction Interrégionale de la mer Méditerranée. Mother colonies were kept separated during larval release by placing each of them in a small 2 L open‐flow aquarium with outlets closed with nylon nets (200 μm pore size) to maintain the water flow and keep the larvae inside the aquarium (Figure 1). Seawater temperature was kept constant between 16°C and 18°C by incubating the small aquaria in a large (100 L) water bath equipped with pumps to ensure continuous mixing of seawater and by keeping the entire system in a temperature‐controlled room (Figure 1). A small fragment (< 2 mm^3^) was removed from the apical branch of each colony and placed in DNA extraction buffer from the direct PCR kit (see below) for microbiome analyses. Three samples of 800 mL seawater surrounding the colony were also collected, filtered onto 0.2 μm Nuclepore Track‐Etch Membrane filters (Whatman, diameter of 47 mm), and the filter retentate was stored in the direct PCR extraction buffer at −20°C until further analyses. For larval collection, care was taken to avoid seawater, which could have contaminated the larvae. Larvae were collected with a Pasteur pipette, rinsed with demineralized water, and placed in 50 μL of the direct PCR extraction buffer.

The life stages of the red coral were sampled under different experimental conditions, allowing for the testing of different symbiont acquisition modes as explained below.

#### 2.1.1 Mode 1: Vertical Transfer of Bacterial Symbionts

During the first day, larvae released by each of the 4 mother colonies were collected immediately after release, identified, and put in DNA extraction buffer for microbiome analyses (Table 1, ‘Larva (newly‐released)’ samples). In such vertical transmission, we kept track of the origin of the larvae mother colonies and determined the proportion that followed this transmission pattern. Only Colony 1 released enough larvae to allow us to assess the early and late vertical acquisition mode of the bacterial symbionts, as described below.

**TABLE 1:** Overview of the different modes and number of samples collected to identify the symbiont acquisition mechanism. One sample of each of the four adult colonies was also collected.

Mode	Symbiont acquisition mode tested	Incubation conditions	Samples collected
1	Early vertical transmission	After spawning: Day 1	2–4 larvae per colony from 3 colonies ‘Larva (newly‐released)’ + 3 samples of seawater ‘Seawater’
2	Early horizontal acquisition	In lab: Days 5 and 10	3 larvae per day at Day 5 and Day 10 from Colony 1 ‘Larva (few days old)’
3	Late horizontal acquisition: seawater and conspecifics	In lab: Month 3 and Year 1	5 settlers of 3‐month‐old age from Colony 1 ‘Settler (3‐month‐old)’ + 4 recruits of 1‐year‐old age from Colony 1 ‘Recruit (1‐year‐old)’
4	Late horizontal acquisition: whole environment	In situ: Year 1 and Year 3	4 recruits of 1‐year‐old age ‘Recruit (1‐year‐old, in situ)’ + 5 juveniles (3‐year‐old) ‘Juvenile’ + 5 samples of sediment ‘Sediment’ + 5 samples from the cave ‘Cave’ + 3 samples of in situ seawater ‘Seawater (in situ)’

#### 2.1.2 Mode 2: Early Horizontal Acquisition of Bacterial Symbionts

Twenty larvae from Colony 1 were transferred to a 2 L‐jar filled with natural seawater, equipped with an air bubbling system, and left in the laboratory to test if larvae acquire their symbionts from the seawater. Thus, larvae were no longer in contact with the mother Colony 1. The larvae from the jar were collected after 5 and 10 days of incubation (Table 1, ‘Larva (few days old)’ samples). Each larva was put in DNA extraction buffer and kept at −20°C until further analyses.

#### 2.1.3 Mode 3: Late Horizontal Acquisition of the Bacterial Symbionts From Conspecifics or Seawater

About thirty larvae released by Colony 1 were transferred in aquaria equipped with an air bubbling system, maintained at a constant temperature (16°C–18°C) and continuously supplied with seawater. Within these aquaria, female and male adult colonies were present, as well as terracotta tiles (20 × 20 cm; immersed in the aquaria 1 month before) to be used as settlement substratum for the larvae. No CCA (often used to induce settlement) were added. After larval settlement on the tiles, 3‐month‐old settlers (Table 1, ‘Settler (3‐month‐old)’ samples) and 1‐year‐old recruits (Table 1, ‘Recruit (1‐year‐old)’ samples) were then collected, put in DNA extraction buffer and kept at −20°C until further analyses.

#### 2.1.4 Mode 4: In Situ Acquisition of Bacterial Symbionts

After recruitment, several tiles from Mode 3 were moved to the artificial caves, containing adult colonies of *C. rubrum* , to assess whether the surrounding environment (i.e., seawater, sediment, other benthic organisms; Table 1) played a role in symbiont transmission. Here, 1‐year‐old recruits (Table 1, ‘Recruit (1‐year‐old, in situ)’ samples) and 3‐year‐old juveniles (Table 1, ‘Juvenile’ samples) were collected along with seawater samples (Table 1, ‘Seawater (in situ)’ samples). Seawater samples (800 mL) were collected in the artificial caves, placed in iceboxes on the diving boat and filtered on 0.2 μm Nuclepore Track‐Etch Membrane filters (Whatman, diameter of 47 mm) at the lab immediately upon arrival. Filters were stored in extraction buffer and kept at −20°C. To characterise the environment within the caves, five 50‐g samples of various colonising benthic species (e.g., a mix of benthic organisms such as algae, sponges and bryozoans) were collected by scraping the caves' walls (Table 1, ‘Cave’ samples), and five samples of sediments were taken from the bottom of the cave (Table 1, ‘Sediment’ samples). These samples (i.e., surrounding environment and sediment) were placed in iceboxes on the diving boat and directly stored at −20°C upon arrival at the lab until further analyses.

### 2.2 Amplification of the *16S rRNA* Gene

A standard approach (i.e., DNA extraction using the Qiagen PowerBiofilm kit followed by PCR amplification of the *16S rRNA* gene) had been tested on individual larvae and recruits, but was unsuitable as DNA yield was too low and PCR amplification did not work (i.e., no trace visible using the Agilent Bioanalyzer DNA 1000 kit; Figure S1). Therefore, a direct PCR approach was chosen to avoid losing the small quantity of (bacterial) DNA present in single larva and recruit samples. Direct PCR has previously been used on various types of samples, including samples with small amounts of DNA (Stojan et al. 2023; Videvall et al. 2017; Cavanaugh and Bathrick 2018; Jung et al. 2024). Here, the direct PCR method was also used on adult coral samples for consistency and to allow comparisons between all coral samples in this study (larvae, settlers, recruits, juveniles, adults).

### 2.3 Coral (Larvae, Recruits, Juveniles and Mother Colonies) and Seawater Samples

After collection, samples were directly put in 50 μL (larva and coral samples) or 100 μL (seawater filters) of extraction buffer of the direct qPCR ProbesMaster kit (Jena Bioscience) to lyse the cells and release DNA. Cell lysis was assisted by adding a Proteinase K solution (0.1 μL; 600 U/mL; Qiagen) and warming the samples at 65°C for 20 min before amplification of the *16S rRNA* gene using the direct PCR. For each sample, three replicate PCRs were done on the V3‐V4 region of the *16S rRNA* gene using the direct qPCR ProbesMaster kit (Jena Bioscience) and the forward and reverse primers 341F 5′‐CCTACGGGNGGCWGCAG‐3′ and 785R 5′‐GACTACHVGGGTATCTAATCC‐3′, respectively (Klindworth et al. 2013). The reaction mixture contained 25 μL of direct qPCR ProbesMaster mix, 0.75 μL of each primer (10 μM), 1 μL of sample (in extraction buffer) and RNAse‐free water to complete the final 50 μL volume. The direct PCR 3‐steps program consisted of an initial step at 95°C for 5 min for enzyme activation, 40 cycles of 15 s at 95°C, 30 s at 55°C and 30 s at 60°C for denaturation, hybridisation and elongation, respectively, then a final step at 60°C for 10 min. For around a quarter of the larva samples, a second attempt was needed for successful PCR amplification. Negative controls (i.e., PCR without sample material) were processed at the same time as the samples. The three PCR reactions per sample were pooled and amplicon purification was done using AMPure XP beads (Beckman Coulter). Amplification and quality of the purified amplicons were checked using the Bioanalyzer DNA 1000 kit (Agilent) (see examples on Figure S1).

### 2.4 Environmental Samples Inside the Artificial Caves (i.e., Surrounding Environment and Sediment)

Fifty grams of each environmental sample were ground to a powder and homogenised. DNA was extracted from 200 mg of each sample using the DNeasy PowerBiofilm kit (Qiagen) with the following modifications: during the cell lysis step, 2 μL of Proteinase K (600 U/mL; Qiagen) was added to the sample and incubated at 60°C for 2 h, followed by 2 min of bead beating using the CryoMill (Retch, Germany) at a frequency of 30 Hz. DNA concentration was measured using an Invitrogen Qubit 4 Fluorometer (Fisher Scientific) and the Qubit dsDNA BR (Broad‐Range) Assay kit, and DNA was stored at −20°C until PCR amplification. PCR conditions were the same as for the other sample types (i.e., coral and seawater) with the exception that only one 30‐cycle PCR reaction (50 μL final volume) was done per sample.

### 2.5 16S rRNA Gene Sequencing and Bioinformatics Data Processing

Purified amplicons were sent to STAB VIDA (Portugal) for amplicon library preparation using Illumina's standard 16S Metagenomic Sequencing Library Preparation protocol (Illumina 2013). Libraries were pooled in equimolar ratios and paired end (2 × 300 bp) sequenced on the Illumina MiSeq platform with V3 chemistry. The fastq files containing the raw sequencing data have been deposited in the NCBI's Short Read Archive (SRA) under the BioProject accession number PRJNA1136805.

MiSeq sequencing produced from 64,960 to 100,010 reads per sample (Table S1). The sequencing of three samples was unsuccessful, and these samples were removed from the dataset (Table S1). The *16S rRNA* gene amplicon data were processed using the DADA2 pipeline (version 1.16) (Callahan et al. 2016). Forward and reverse reads were trimmed and filtered with the following settings: truncLen = c(260, 220), maxN = 1, maxEE = c (Bemark et al. 2024), rm.phix = TRUE, trimLeft = c (Chamberland et al. 2017; Nitschke et al. 2016). Error rates were computed and used for sequence inference. Resulting forward and reverse reads were then merged, and those smaller than 390 bp and longer than 450 bp were removed. Sequences were checked for chimeras that were removed. An amplicon sequence variant (ASV) count table was created containing 6782 ASVs. The number of reads passing the different steps of the pipeline per sample are presented in Table S1. Taxonomy was assigned to each ASV using the RDP naive Bayesian classifier method, the SILVA SSU reference database (version 138.1) and a minimum bootstrap confidence of 50. ASVs classified as ‘Chloroplast’ were removed from the dataset. The ASV table, the ASV taxonomy, the sequences of each ASV, and the metadata are available in (Tables [Link], [Link]).

### 2.6 Data Analyses

Graphical and statistical analyses were carried out in the R environment (version 4.2.2). The R‐package *decontam* was used to identify potential contaminant ASVs in the samples based on the negative control samples (*isContaminant* function) (Davis et al. 2018). Thirty‐eight ASVs were identified as contaminant and then removed. Negative control samples were excluded from the dataset prior to further analyses. Potential differences in observed richness (number of ASVs per sample) were explained by constructing a generalised linear model (GLM) using a negative binomial distribution, considering the discrete and over‐dispersed nature of the richness data. The different life stages of the red coral were considered as fixed factors (Table S5; randomly rarefied community data to the lowest read number). For statistical analyses, the 5‐ and 10‐day old larva samples were combined into a new group because of too few replicates. This new group including all the 5‐ and 10‐day old larva samples are named ‘few days old larvae’ throughout the manuscript. To examine changes in the composition of *C. rubrum* microbiome at the ASV and family level, we used a compositional data analysis (CoDA) approach (Gloor et al. 2017). Raw ASV abundances were transformed by calculating the centred log‐ratios (clr) as implemented in the R‐package *compositions* (van den Boogaart and Tolosana‐Delgado 2008), after imputing zero counts based on Bayesian multiplicative replacement (Bayes‐LaPlace BM method of the *cmultRepl* function of the R‐package *zCompositions*) (Palarea‐Albaladejo and Martín‐Fernández 2015). Clr‐transformed count data were then used as inputs for multivariate hypothesis testing. An Aitchison distance matrix was generated by calculating the Euclidean distances between samples based on the clr‐transformed data table. Based on the Aitchison distance matrix, principal component analyses (PCA), as well as permutational multivariate analysis of variance (PERMANOVA/adonis from the R‐package *vegan* and permanova_pairwise from the R‐package *ecole* adjusting the *p* values with the false discovery rate correction; 999 permutations; (Oksanen et al. 2015; Smith 2021)) and dispersion analyses (PERMDISP; R‐package *vegan*) were used to assess differences in bacterial community diversity between the different life stages of *C. rubrum* and between the life stages and the environment.

## 3 Results

### 3.1 Bacterial Species Richness in the Early Life Stages of *C. rubrum*

**FIGURE 2:**
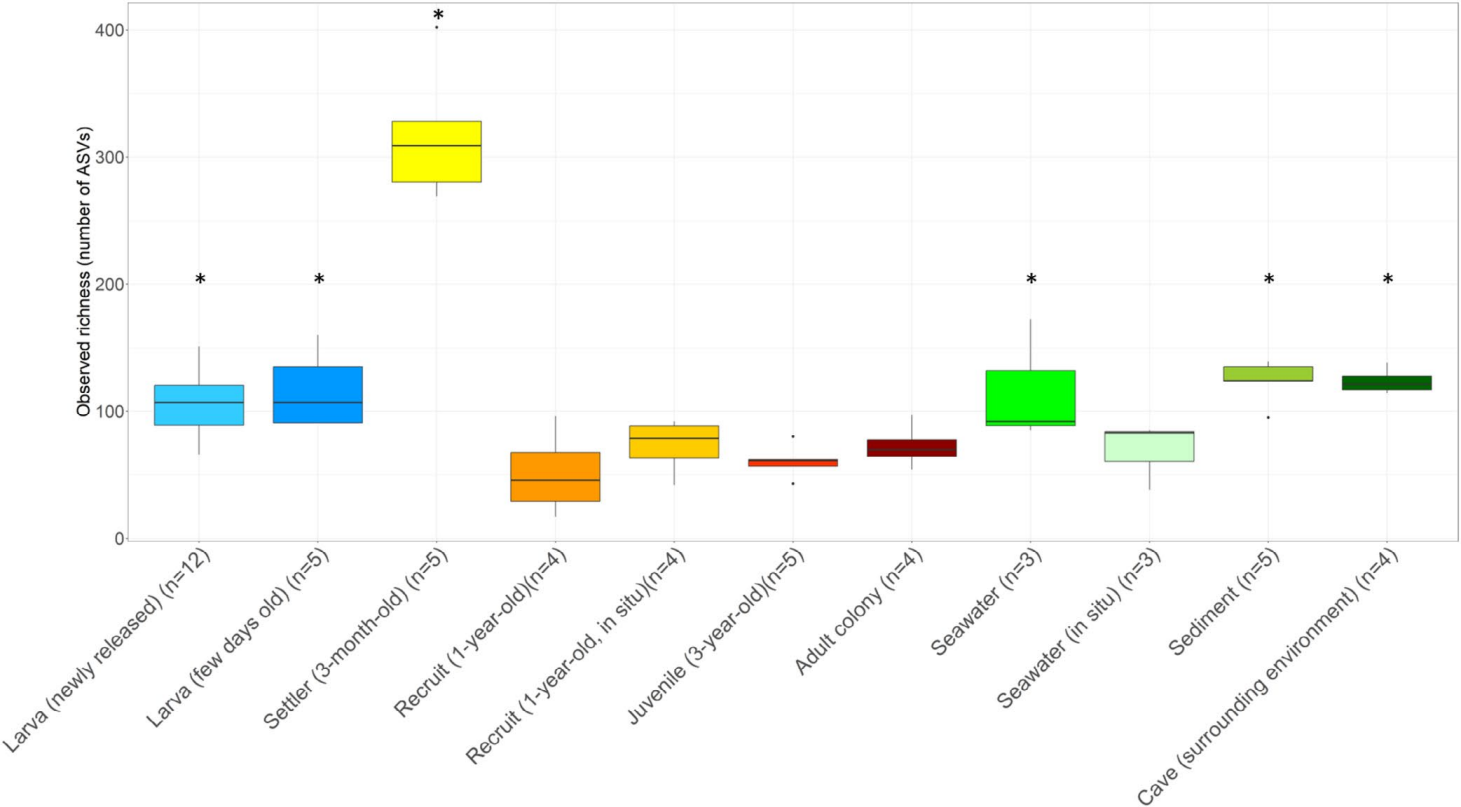
Bacterial species richness in the different life stages of *C. rubrum* and the nearby environment. Sampling conditions for the different samples are found in Table 1. ‘*n*’ corresponds to the number of replicates per category. Statistical significance levels of differences compared to adult colonies are indicated by asterisks (* for *p* < 0.05).

On average, the estimated bacterial richness was lower in adult colonies than in the newly released larvae, the few days old larvae, and the 3‐month‐old settlers (glm.nb *p* = 0.009, 0.005 and 2 × 10^−16^, respectively; Figure 2, Tables S1 and S5). No differences were observed between the 1‐year‐old recruits, the 3‐year‐old juveniles and adult colonies, as well as between the 1‐year‐old recruits grown in situ and in the laboratory (*p* > 0.05; see Table S5 for pairwise comparisons). The estimated bacterial richness was higher in the environmental samples (i.e., sediment, and surrounding environment or cave) and the laboratory seawater than in adult colonies (Figure 2, Tables S1 and S5).

### 3.2 Bacterial Community Composition in the Different Life Stages

**FIGURE 3:**
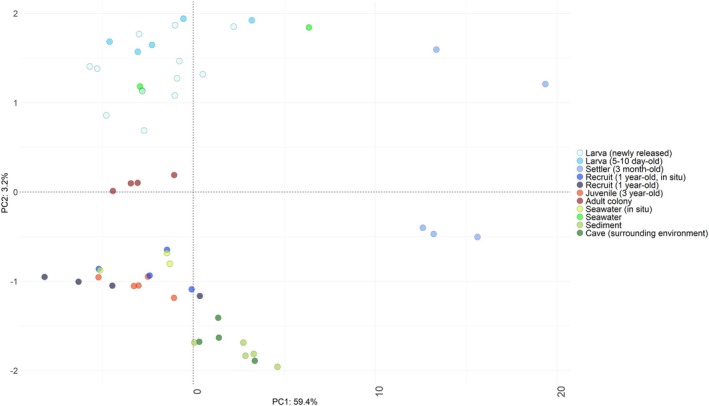
Beta diversity of the bacterial community associated with the different life stages of *C. rubrum* and found in the nearby environment. Principal component analysis based on the Aitchison distance matrix showing the distribution and dispersion of the samples (ASV level). Sampling conditions for the different samples are found in Table 1.

Overall, the composition of the bacterial community was structurally different between the different life stages and between the life stages and the environmental samples (PERMANOVA *p* < 0.05; ASV level; see Table S6 for pairwise comparisons), except between the newly released larvae and the few days old larvae, and between the juveniles and the 1‐year‐old settlers (*p* = 0.36; *F* = 0.9, and *p* = 0.32; *F* = 1.1, respectively; Figure 3, Figure S2 and Table S6). Three‐month‐old settler samples were more dispersed compared to the other life stages (Table S7; *p* values < 0.05; ASV level). The structure of the bacterial community associated with the larvae was different from the structure of the seawater bacterial community (PERMANOVA *p* = 0.03 *F* = 3.3 and *p* = 0.01 *F* = 4.3 for the newly‐released larvae and few‐days‐old larvae, respectively; ASV level; Table S6).

**FIGURE 4:**
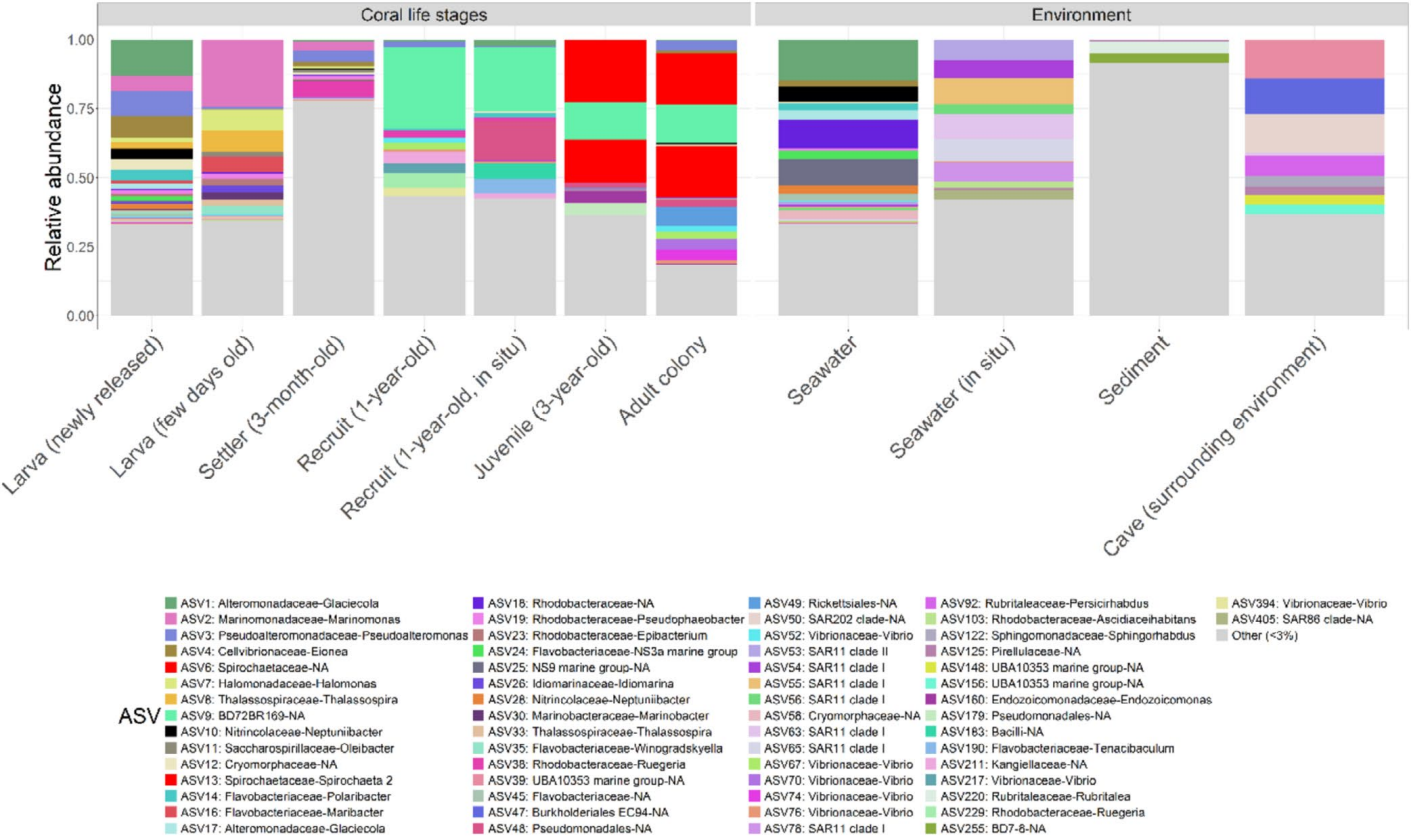
Relative abundance of the bacteria associated with the different life stages of *C. rubrum* and found in the nearby environment (average relative abundances; ASV level). ASVs whose relative abundance is < 3% are grouped in ‘Other’. Sampling conditions for the different samples are found in Table 1.

Over the course of the first year, the most abundant bacterial ASVs differed between life stages. Newly released larvae harboured primarily ASV1‐*Alteromonadaceae Glaciecola* (this ASV represents 13% ± 9% of the community), ASV3‐*Pseudoalteromonadaceae Pseudoalteromonas* (8% ± 14%) and ASV4‐*Cellvibrionaceae Eionea* (7% ± 10%), whereas the microbiota of the few days old larvae were dominated by ASV2‐*Marinomonadaceae Marinomonas* (24% ± 7%), ASV8‐*Thalassospiraceae Thalassospira* (8% ± 5%) and ASV7‐*Halomonadaceae Holomonas* (7% ± 3%). The bacterial communities associated with recruits contained mostly ASV38‐*Rhodobacteraceae Ruegeria* (6% ± 3%), ASV3‐*Pseudoalteromonadaceae Pseudoalteromonas* (5% ± 7%) and ASV2‐*Marinomonadaceae Marinomonas* (2% ± 3%) 3 months after spawning, but ASV9‐*BD72BR169* (30% ± 31%), ASV229‐*Rhodobacteraceae Ruegeria* (5% ± 7%) and ASV211‐*Kangiellaceae* (5% ± 6%) after 1 year in aquarium conditions. This differed from in situ conditions, where 1‐year‐old recruits harboured predominantly ASV9‐*BD72BR169* (23% ± 6%; *Gammaproteobacteria*), ASV48‐*Pseudomonadales* (12% ± 11%) and ASV183‐*Bacilli* (5% ± 9%). The dominant ASVs in juvenile (3 years old) and adult colonies were respectively the same: ASV6‐*Spirochaetaceae* (23% ± 19% and 18% ± 21%), ASV13‐*Spirochaetaceae* (16% ± 11% and 20% ± 24%) and ASV9‐*BD72BR169* (*Gammaproteobacteria*; 13% ± 11% and 14% ± 21%) (Figures 4 and S3). Among the 5 most abundant ASVs present in adult colonies, ASV6 and ASV13, both *Spirochaetaceae*, and ASV49‐*Rickettsiales* were only detected in the 3‐year‐old colonies; ASV9‐*BD72BR169* (*Gammaproteobacteria*) was detectable in the 1‐year‐old recruits and the 3‐year‐old colonies, and finally ASV3‐*Pseudoalteromonadaceae Pseudoalteromonas* was detected in all life stages as well as in very low abundance in the seawater samples (0.03% ± 0.04%; Figure 4).

## 4 Discussion

The present study has significantly improved our understanding of the dynamics and timing associated with the acquisition of the microbiota in *C. rubrum* . For the first time, a comprehensive analysis of the bacterial composition was conducted for a Mediterranean octocoral across different life stages, from newly released larvae to 3‐year‐old juvenile colonies. Our findings indicate that the predominant symbionts of adult colonies are absent in larvae and in recently settled individuals. Some of these symbionts (e.g., *BD72BR169 Gammaproteobacteria*) only emerge between 6 months and one year of age, whereas *Spirochaetaceae*, which are highly dominant in the microbiota of adult colonies, were found only in 3‐year‐old colonies. Their appearance in the *C. rubrum* microbiota after 6 months (or longer) shows that these bacteria play no role in the initial stages of colony development. Instead, their presence seems to become relevant as the colonies mature, possibly contributing to the stability and functionality of the adult microbiota. These results provide new insights into the evolution of microbial associations in *C. rubrum* and emphasise the possibility of stage‐specific microbiota that evolve as the host progresses through different life stages.

### 4.1 Horizontal Acquisition of Bacterial Symbionts

Symbiont acquisition in corals is independent of the mode of sexual reproduction, and in both brooders and broadcast spawners, the transmission mechanism of symbionts can be either vertical or horizontal (Quigley et al. 2018; Baird et al. 2021; Quigley et al. 2017). *C. rubrum* has a brooding reproductive strategy, and it had thus been suggested that the transmission of the bacterial symbionts may be vertical, from the mother colonies to the larvae (van de Water, Allemand, et al. 2018). However, looking at the 5 most abundant symbionts found in the adult colonies, 3 were detected in the 3‐year‐old juveniles (*Spirochaetaceae* ASV6 and ASV13, and *Rickettsiales* ASV49), and only one was found in both the 1‐year‐old recruits and the 3‐year‐old juveniles (*Gammaproteobacteria BD72BR16*, ASV9). Altogether, these results suggest that the acquisition of these bacteria is horizontal (from the environment), and not vertical, as it was previously suggested (van de Water, Allemand, et al. 2018). In contrast, ASV3‐*Pseudoalteromonadaceae* was present in all life stages and could have been acquired either vertically via the parents or horizontally from the water.

Horizontal transmission requires the uptake of microbes from the environment. Apart from *Pseudoalteromonadaceae*, the other dominant symbionts were not detected in the environmental samples collected, that is, in the seawater, in the biological material surrounding the colonies in the underwater cave, and in the sediment. However, *Spirochaetaceae* bacteria have been found and isolated from marine sediments, including in the Mediterranean Sea (Subhash and Lee 2017; Aldeguer‐Riquelme et al. 2022; Tamburini et al. 2020; Bech et al. 2020; Miyazaki et al. 2014). The horizontal transmission of the bacterial symbionts could also be facilitated by nearby organisms (Hochart et al. 2024). As the larvae of *C. rubrum* generally settle very close to the mother colonies (Costantini et al. 2016; Ledoux et al. 2010), the most likely source would be nearby adult colonies that shed microbes to regulate their abundances in the new holobionts (Garren and Azam 2012). This would also explain the geographical stability of the microbiota of *C. rubrum* (van de Water et al. 2016). Another potential source may be the CCAs (Hochart et al. 2024), which are the main builders of coralligenous habitat and are known to attract and promote the settlement and metamorphosis of larvae of several coral species (Jorissen et al. 2021; Gómez‐Lemos et al. 2018; Kitamura et al. 2009; Tebben et al. 2015; Petersen et al. 2021; Siboni et al. 2020; Kitamura et al. 2007). Field observations and experimental studies have shown interactions between *C. rubrum* larvae and CCA (sympatry and facilitation during larval settlement (Toma et al. 2022; Zelli et al. 2020)). The mechanisms of larval settlement (i.e., preference for certain CCAs and possible preference of conspecifics) ensure that also in case of long dispersal, they will settle in an environment where they can find the correct microbiota. Although horizontal transmission involves uncertainty as to whether suitable symbiotic partners will meet in subsequent generations, this mechanism might be advantageous at the evolutionary scale, as corals interact with a large variety of symbionts, potentially facilitating adaptation to environmental conditions. It is therefore striking to see how stable the microbiota of adult *C. rubrum* is on temporal and geographical scales (van de Water et al. 2016; van de Water, Voolstra, et al. 2018), and across studies (Tignat‐Perrier et al. 2023; Tignat‐Perrier et al. 2022; Prioux et al. 2023).

### 4.2 Late Maturation of the Microbiota of *C. rubrum*

Over the course of the first few years of their life cycle, *C. rubrum* colonies underwent strong changes in the richness and the structure of their associated bacterial communities. According to our results, microbiota structure changes during colony development and estimated bacterial richness decreases, being on average higher in the early life stages than in adults, which is similar to what has been observed in other coral species (Quigley et al. 2018; Damjanovic et al. 2020a; Damjanovic et al. 2020b). For example, Damjanovic et al. (2019) observed a higher diversity in the bacterial community composition between larvae, 4‐day‐old settlers, and adults in the tropical coral *Pocillopora acuta* (Damjanovic et al. 2020b). It has been observed that even the composition of the bacterial community associated with the embryonic developmental stages of the scleractinian coral *Acropora tenuis* , that is, the oocyte‐sperm bundle, two‐cell (3 h‐old), prawn chip (12 h‐old), gastrula (36 h‐old) and larval (96 h‐old) stages, differed (Damjanovic et al. 2020a). In particular, members of *Oceanospirillaceae* were mostly present in the gastrula and larval stages, while several *Rhodobacteraceae*, *Cryomorphaceae* and *Cellvibrionales* were indicators for the two‐cell and prawn chip stages (Damjanovic et al. 2020a). The higher bacterial richness observed in the early life stages of the red coral may be attributed to the constant exposure of the larvae to seawater microbes immediately following their release. Although we took care to minimise the amount of seawater collected with the larvae and rinsed them with demineralized water, completely avoiding it was challenging due to the small size of the larvae (approximately 1 mm). After metamorphosis, the resulting settlers—primary polyps—come into direct contact with the substrate, which may introduce additional microbial interactions from the surrounding rocky surface. The duration and nature of these interactions with environmental microbes likely vary. Some microbes may be transiently associated, potentially representing contaminants, while others may establish more persistent relationships and evolve into true symbionts.

*Pseudoalteromonadaceae* (ASV3‐*Pseudoalteromonas*) were detected in relatively high abundance in all life stages, as well as in the surrounding environment. Members of this family are commonly found in various marine environments, including seawater, sediment and in association with organisms, such as algae and invertebrates (Ivanova et al. 2014; Holmström and Kjelleberg 1999). Although their function(s) in *C. rubrum* are unknown, some *Pseudoalteromonadaceae* bacteria associated with tropical corals have been shown to produce bioactive compounds with antimicrobial, antifouling and cytotoxic properties (Shnit‐Orland et al. 2012; Sweet et al. 2021). Some also produce tetrabromopyrrole, which stimulates metamorphosis in a range of coral species (Tebben et al. 2011; Negri et al. 2001; Sneed et al. 2014). Therefore, *Pseudoalteromonadaceae* may also be involved in *C. rubrum* larval metamorphosis and may play a role in regulating the microbiota of larvae and recruits.

*Spirochaetaceae* and the *Gammaproteobacteria BD72BR169*, which together dominate the microbiota of adult colonies, were not detectable in the larvae and 3‐month‐old settlers. *BD72BR169 Gammaproteobacteria*, however, appeared in 1‐year‐old recruits and thus colonised *C. rubrum* before the *Spirochaetaceae* bacteria. They were detectable in individuals reared both in situ and in the laboratory, suggesting that their reservoir might be the conspecific adult colonies. Other dominant bacteria (e.g., ASV211‐*Enterobacterales*) in the 1‐year‐old recruits differed in relative abundance between the laboratory and in situ conditions and were not present in the 3‐year‐old colonies anymore. For other coral species, it has been hypothesised that a selection process occurs during early ontogeny, in which the coral gradually fine‐tunes its microbiota until a more stable and restricted community establishes (Leite et al. 2017; Zanotti et al. 2020; Damjanovic et al. 2020a; Damjanovic et al. 2020b). The immaturity of the microbiota in the early life stages, indicated by the continuous changes in composition, suggests a similar process for *C. rubrum* . The late acquisition of *Spirochaetaceae* and *BD72BR169 Gammaproteobacteria* thus suggests that they do not play a significant role in the development of larvae and juveniles.

*Spirochaeta*, belonging to the *Spirocheataceae*, were recently suggested to play a role in the red coloration of *C. rubrum* 's tissues and skeleton (van de Water et al. 2024). In this study, we noticed, however, that the skeletons of settlers and recruits were already red‐coloured (Figure 1C for the 1‐year‐old recruits). As *Spirochaetaceae* are not yet present in the holobiont of *C. rubrum* at these life stages, their role in this coral's colour may thus be rather limited, or even non‐existent.

Determining the functions of the symbionts in the adult stages would help to understand their contribution to the functioning and health of *C. rubrum* 's holobiont. For certain coral species, the parental transmission and systematic presence of bacteria (i.e., obligate relationship) from larvae to coral juveniles suggest a functional importance for larval development (Engelberts et al. 2022). In the spawning corals *A. millepora* , *A. digitifera* and *P. acuta*, for example, *Rhodobacteraceae* bacteria were consistently present in larvae, suggesting a parental transmission and a possible key role of the symbionts in early life stages (Damjanovic et al. 2020a, 2020b; Bernasconi et al. 2019). However, only assumptions have been made for now, and the role of vertically acquired bacterial symbionts in coral ontogeny remains to be defined.

### 4.3 Direct PCR for Metabarcoding of Low Biomass Samples

We used a novel direct PCR‐based method to generate *16S rRNA* gene amplicons for metabarcoding. The direct PCR applied to adult corals yielded a similar microbial community—and the dominance of *Spirochaetaceae* symbionts—as the more usual method (van de Water et al. 2016; van de Water, Voolstra, et al. 2018; Tignat‐Perrier et al. 2023). This approach was chosen to study the microbiota of individual larvae and recruits, which is crucial for studying the transmission of symbionts to offspring. The very small size of coral larvae and recruits makes it difficult to analyse the diversity of their microbiota as the biomass and thus the quantity of DNA is too low, impairing bacterial DNA extraction. In previous studies, coral larvae were therefore pooled to obtain sufficient bacterial DNA for subsequent PCR amplification of *16S rRNA* gene amplicons after total DNA extraction and purification (e.g., 50–70 pooled larvae in (Damjanovic et al. 2020a); 10 pooled larvae in (Zanotti et al. 2020); 20 pooled larvae in (Lema et al. 2014)). As the direct PCR‐based method does not require a separate DNA extraction step, potential DNA loss is avoided and the risk of contamination with foreign DNA is limited. It should be noted, however, that several attempts at PCR amplification may still be needed in order to obtain sufficient amounts of amplicons for sequencing library preparation (as it was the case for around a quarter of the larval samples). Although the method could be further optimised, direct PCR might be a suitable method to analyse the microbiota of coral larvae using *16S rRNA* gene amplicon metabarcoding.

## 5 Conclusion

Our study sheds light on the bacterial colonisation and microbiota maturation of *C. rubrum* holobionts from early life stages to adulthood. Our observations suggest that the establishment of the adult coral microbiota takes at least 1 year. During this time, a variety of bacteria meets the coral host, but only some of them are then selected to be part of the adult holobiont. Further studies could help to understand the mechanisms of this selection and the possible role of environmental conditions, sympatric species and coral needs. Our results showed that the dominant symbionts of the adult colonies (i.e., *Spirochaetaceae* and the *BD72BR169 Gammaproteobacteria*) are acquired from the environment between 6 months and 3 years of age, but the source remains unclear, as these ASVs were not found in the environmental samples. While the role of these symbionts within the *C. rubrum* holobiont remains unclear, our results suggest that they probably do not play a major role during early ontogeny. To gain further insights into the predominant symbioses of *C. rubrum* , we need to address the mechanism by which symbioses between the coral host and Spirochaetaceae and other bacteria develop, and how symbionts are selected to ultimately obtain the characteristic and stable bacterial community of adult *C. rubrum* colonies.

## Author Contributions

**R. Tignat‐Perrier:** conceptualization, methodology, data curation, investigation, validation, formal analysis, visualization, writing – original draft. **L. Bramanti:** conceptualization, investigation, methodology, writing – review and editing. **B. Giordano:** investigation, methodology, writing – review and editing. **J. A. J. M. van de Water:** methodology, writing – review and editing. **E. Manea:** investigation, writing – review and editing. **D. Allemand:** funding acquisition, supervision, writing – review and editing. **C. Ferrier‐Pagès:** conceptualization, supervision, writing – original draft.

## Conflicts of Interest

The authors declare no conflicts of interest.

## Supporting information


**Figure S1.** Verification of the PCR amplification of the *16Sr RNA* gene using the Agilent Bioanalyzer DNA 1000 kit. (A) 2 examples of traces of larval samples; (B) 2 examples of traces of adult coral samples; (C) 2 examples of traces of negative control samples (direct PCR method).
**Figure S2.** Beta diversity of the bacterial community associated with the different life stages of *C. rubrum* and found in the nearby environment. Principal component analysis based on the Aitchison distance matrix showing the distribution and dispersion of the samples (family level). Sampling conditions for the different samples are found in Table 1.
**Figure S3.** Relative abundance of the bacteria associated with the different life stages of *C. rubrum* and found in the nearby environment (average relative abundances). (A) Bacterial families and (B) ASVs whose relative abundance is > 3%. Sampling conditions for the different samples are found in Table 1.

**Table S1.** Metadata.
**Table S5.** GLM analysis on the richness (pairwise analysis).
**Table S6.** Pairwise permanova on the diversity (fdr p.adj).
**Table S7.** Results of the betadispersion analysis.

**Table S2.** ASV table (raw abundances).

**Table S3.** ASV sequences.

**Table S4.** ASV taxonomy.

## Data Availability

The data that support the findings of this study are openly available in NCBI's Short Read Archive at https://www.ncbi.nlm.nih.gov/sra, reference number PRJNA1136805.
