# Supplementary material for: Microbiome Dynamics in Early Life Stages of the Precious Mediterranean Red Coral Corallium rubrum
Source: Environ Microbiol Rep. 2025 Jun 17;17(3):e70127. doi: 10.1111/1758-2229.70127 (PMC12172809; doi:10.1111/1758-2229.70127)
Supplement: Supplementary file 1 — Figure S1. Verification of the PCR amplification of the 16Sr RNA gene using the Agilent Bioanalyzer DNA 1000 kit. (A) 2 examples of traces of larval samples; (B) 2 examples of traces of adult coral samples; (C) 2 examples of traces of negative control samples (direct PCR method). Figure S2. Beta diversity of the bacterial community associated with the different life stages of C. rubrum and found in the nearby environment. Principal component analysis based on the Aitchison distance matrix showing the distribution and dispersion of the samples (family level). Sampling conditions for the different samples are found in Table 1. Figure S3. Relative abundance of the bacteria associated with the different life stages of C. rubrum and found in the nearby environment (average relative abundances). (A) Bacterial families and (B) ASVs whose relative abundance is > 3%. Sampling conditions for the different samples are found in Table 1. [file EMI4-17-e70127-s005.docx]

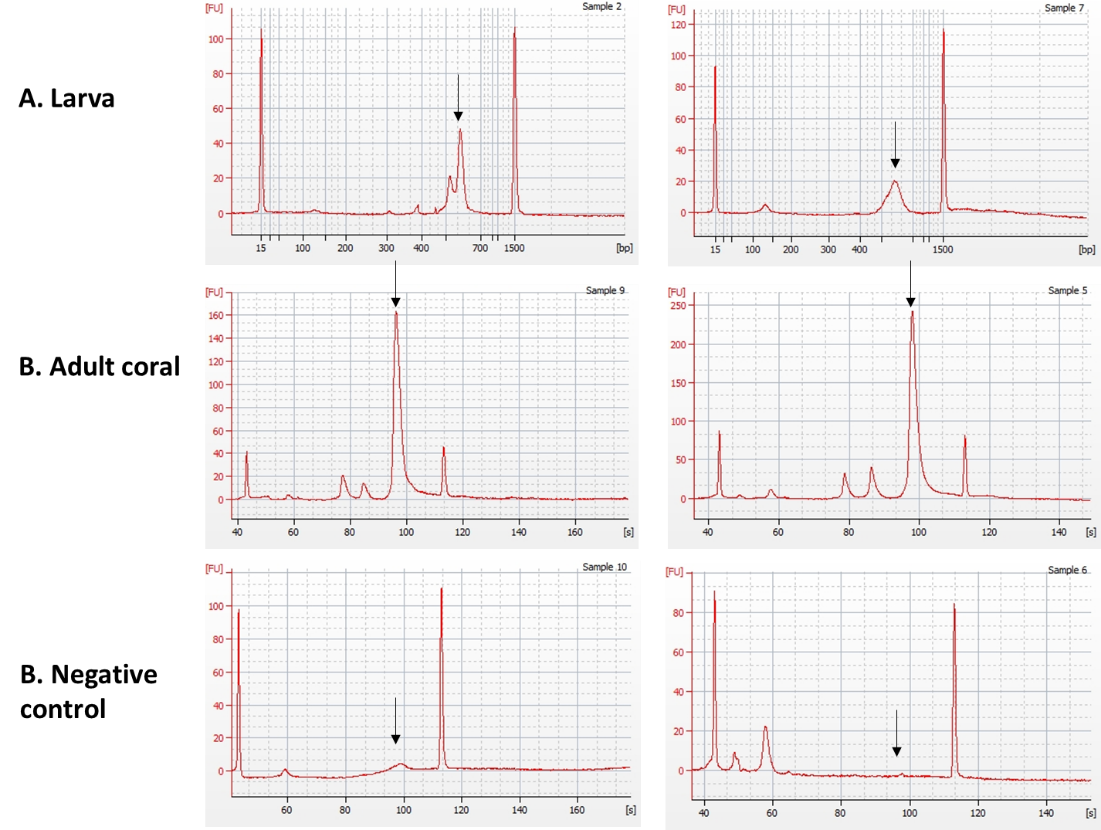


Fig S1. Verification of the PCR amplification of the *16Sr RNA* gene using the Agilent Bioanalyzer DNA 1000 kit. **A.** 2 examples of traces of larval samples; **B.** 2 examples of traces of adult coral samples; **C.** 2 examples of traces of negative control samples (direct PCR method).


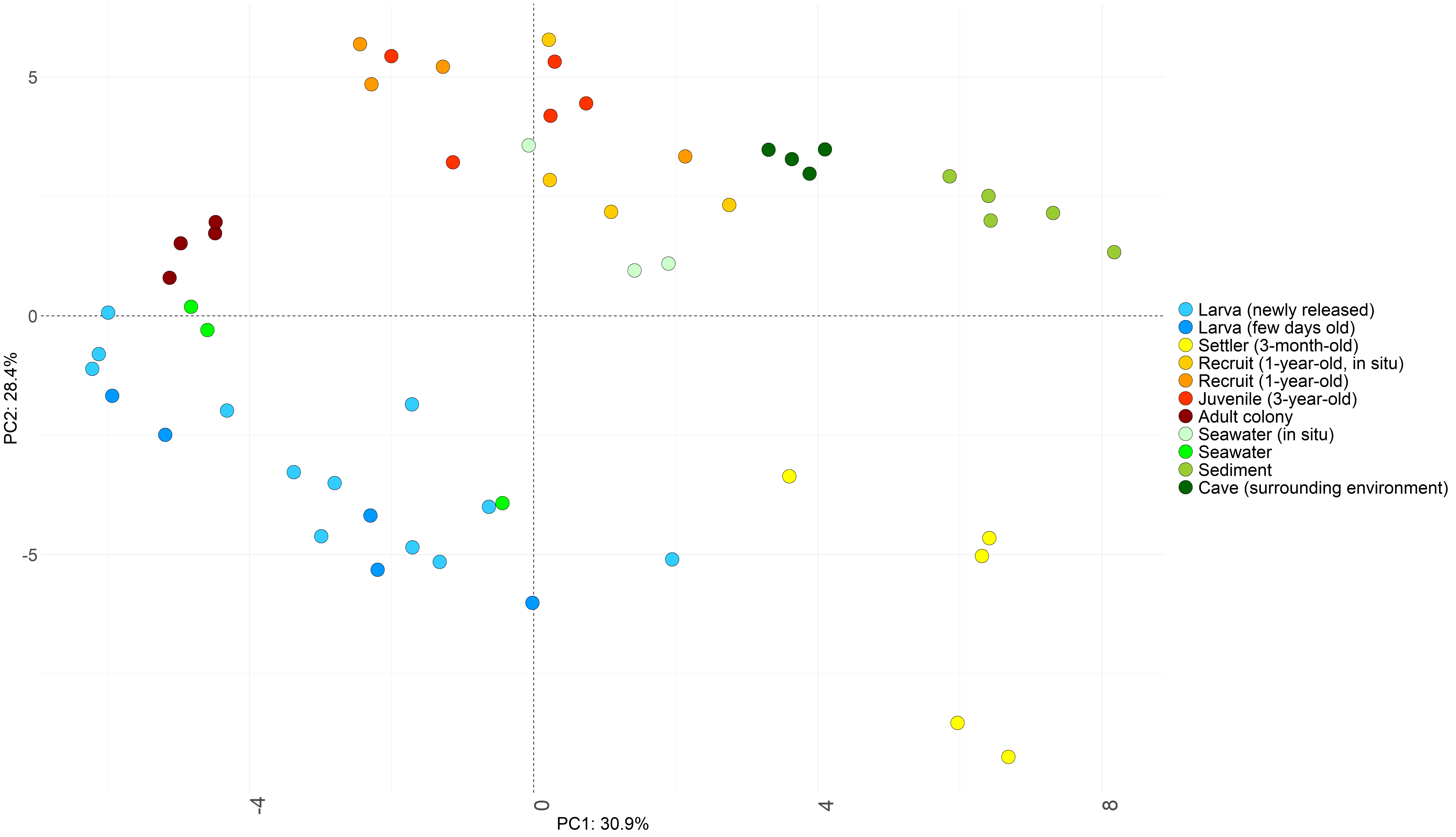


Fig. S2. Beta diversity of the bacterial community associated with the different life stages of *C. rubrum* and found in the nearby environment. Principal component analysis based on the Aitchison distance matrix showing the distribution and dispersion of the samples (family level). Sampling conditions for the different samples are found in Table 1.

(***A***)


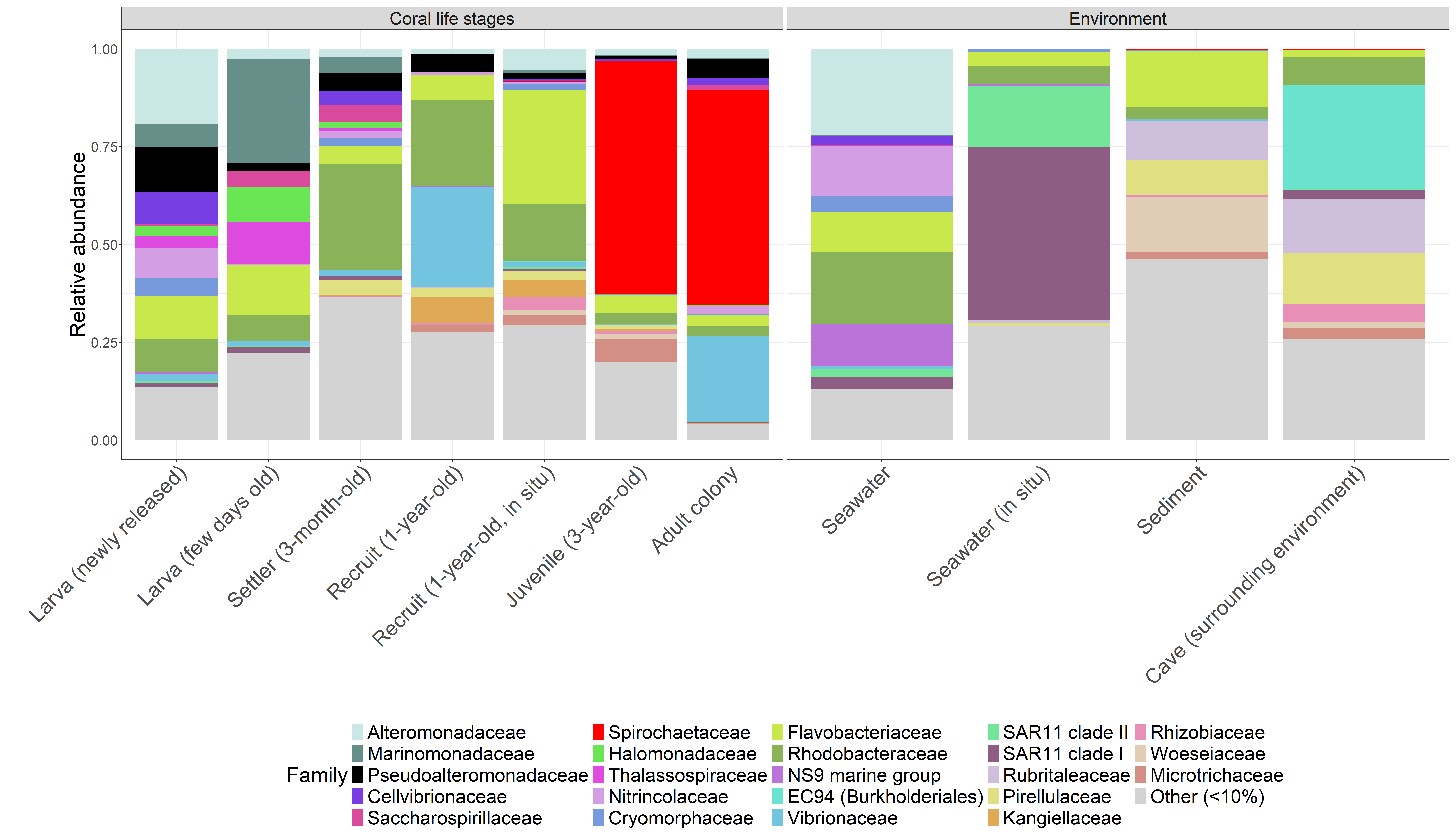


(***B***)


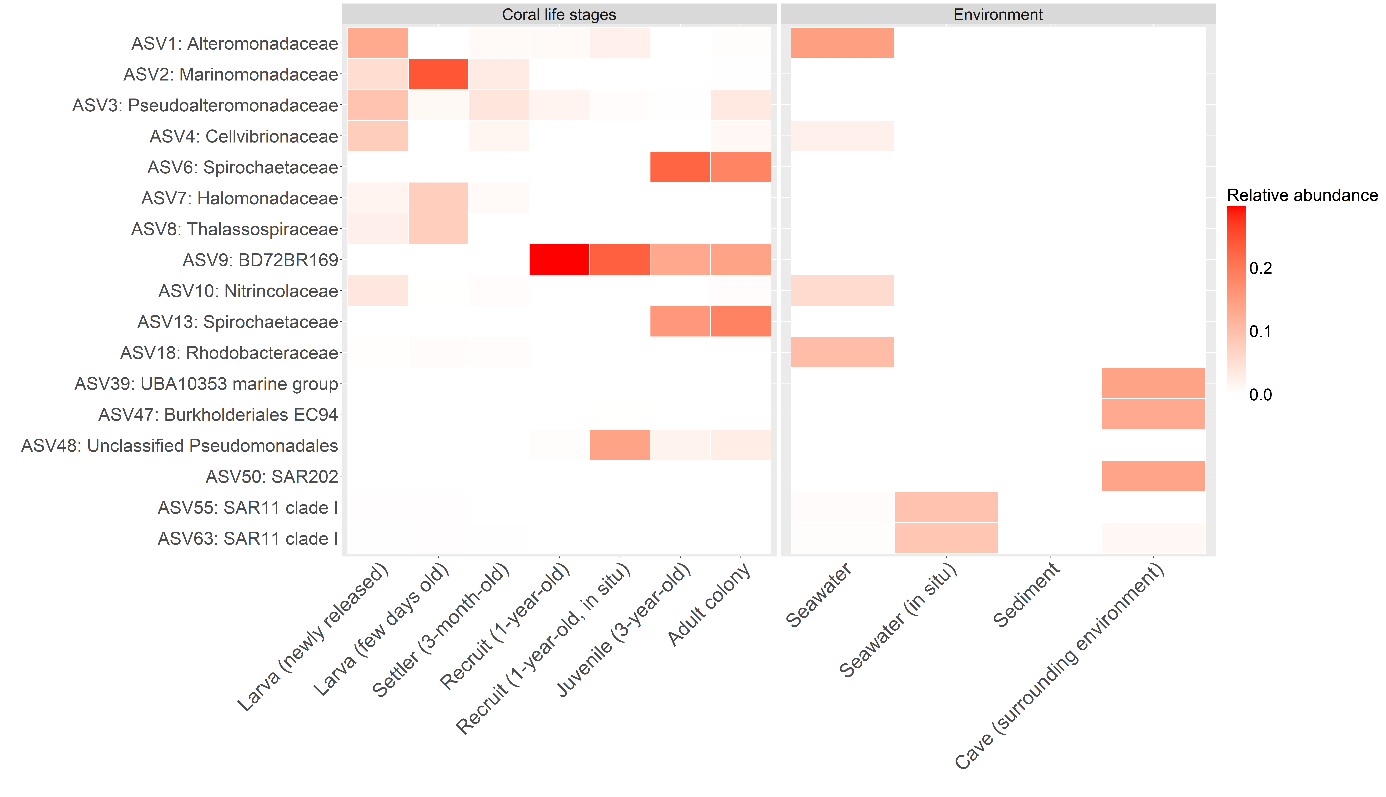


Fig S3. Relative abundance of the bacteria associated with the different life stages of *C. rubrum* and found in the nearby environment (average relative abundances). (***A***) Bacterial families and (***B***) ASVs whose relative abundance is > 3%. Sampling conditions for the different samples are found in Table 1.
